# The US war on harm reduction: fixing policy on intelligence and facts

**DOI:** 10.1186/1477-7517-2-14

**Published:** 2005-09-15

**Authors:** Alex Wodak

**Affiliations:** 1 Director, Alcohol and Drug Service, St Vincent's Hospital, Sydney, Australia; 2 Conjoint Senior Lecturer, School of Public Health and Community Medicine, School of Medicine, The University of New South Wales, Sydney, Australia

At a two-day private meeting in Tokyo in June 2005, some of Japan's most senior politicians and powerbrokers met to consider the steadily expanding HIV/AIDS epidemic. AIDS has recently become a matter of increasing concern in Japan following an HIV epidemic in several major Japanese cities among Japanese men having sex with men at sex-on-premises venues. The Japanese elites at the Tokyo meeting were shocked to learn that the United States has by far the highest annual AIDS incidence among OECD countries at 15/100,000 [1]. Spain, with an annual AIDS incidence of 3.3/100,000, has the second highest rate among industrialized countries, while Australia was well down the ranking with an incidence only one tenth that of the United States at 1.5/100,000 [1].

The pragmatic Japanese were stunned to learn that the high AIDS incidence in the United States was no accident: abstinence-only rather than explicit, peer-based sex education and tokenistic, rather than early and vigorous, needle syringe programmes have produced the expected public health outcomes. In 2002, needle syringe programmes in the United States actually declined from the previous year, exchanging and distributing 25 million needles and syringes [2] for a total population of about 290 million. In contrast, Australia, with a population of 20 million, exchanged and distributed 32 million needles and syringes in 1998/99 [3]. As Randy Shiltz recorded in 'And The Band Played On' [4], from the outset the United States responded to the greatest global public health crisis of the last half millennium with consistent and breath-taking denial. President Reagan failed to make any public comment on HIV/AIDS for the first six years of the epidemic. Three Presidents later, little has changed. It's still business as usual despite the United States failing to meet government declared targets for reducing the number of new HIV infections.

The HIV/AIDS epidemic was officially recognized almost a quarter century ago. In that time, the intelligence and facts about prevention of HIV have become well established. What is less well appreciated is the pivotal importance of political leadership in translating the intelligence and facts about prevention into evidence-based programmes established in time and on a scale commensurate with control of the epidemic. It was political leadership in Uganda, Thailand and Cambodia which changed the trajectory of three epidemics based on high partner change heterosexual activity. Political leadership in Australia, in partnership with community activists, clinicians and researchers, tamed the early HIV epidemic among men who have sex with men and averted an epidemic among injecting drug users. Likewise, the political leadership provided from within the Thatcher government in Great Britain ensured that an HIV epidemic among and from injecting drug users was averted by pragmatism. But political leadership in the United States of America not only deprived the citizens of that country of the benefits of evidence-based HIV prevention, but also actively exported these failed policies to other countries. Nowhere has this been clearer than in any HIV prevention policy or programme linked to injecting drug users.

At the very same time as the meeting discussed above took place in Tokyo, 22 nations attended the Programme Coordinating Board of UNAIDS in Geneva to finalise a policy paper on HIV prevention [5]. The United States had insisted during the twelve month development of the document that phrases such as 'harm reduction' and 'needle syringe programmes' must be excluded. In the June 2005 Geneva meeting, the Indian delegation noted that India and the United States of America were the world's two largest democracies and asked the delegation of the United States to respect the weight of world opinion: none of the 21 other countries supported the position of the United States. After two days of difficult discussion, the United States grudgingly allowed these (and other similarly pragmatic) phrases to be included.

Only three months earlier, at the United Nations Commission on Narcotic Drugs meeting in Vienna in March 2005, a similar debate took place. On that occasion, the United States, with the support of Japan and Russia, was able to hold out its abstinence-only position against 17 other countries who wanted the CND document to explicitly support harm reduction.

In the last few years, most of the major countries in Asia have come to realize that harm reduction policies and programmes are critical to control of HIV among and from injecting drug users. China, Vietnam, Malaysia, Indonesia, Burma and Taiwan are all now traveling down the same road. They all started as zealous supporters of a law enforcement dominated approach to drugs, and are all now moving to a more pragmatic and evidence based public health approach in which HIV control can be achieved. Methadone and needle syringe programmes are planned or already being established in these countries. Contradictions between the new harm reduction approach and the former law enforcement dominated approach are being recognized and dealt with. Thailand is now isolated as the last major Asian country to still support a scorched earth War on Drugs.

The exceptionalism of the United States, discussed since the time of Alexis de Tocqueville, has been increasing in recent years, especially since the election in 2000 of President George W Bush [6]. The United States of America is becoming increasingly isolated, not only among other developed countries, but also in the developing world.

On May 1, 2005, The Sunday Times in England published a leaked document [7] which is accepted as the official minutes of a meeting held at 10 Downing Street on 23 July 2002 to enable 'C' (Sir Richard Dearlove), then head of MI 6, to report to the British Prime Minister and his senior Cabinet colleagues and major government officials. The subject was a briefing 'C' had just received in Washington from George Tenet, then head of the CIA, regarding the forthcoming invasion of Iraq. Among the astonishing revelations in these minutes is the comment by 'C' 'but the intelligence and facts were being fixed around the policy.' The inescapable conclusion from reading these minutes is that Tenet advised 'C' that the intelligence and facts on Iraq were being adjusted in the United States to justify the decision to invade Iraq. While the revelations in these minutes have surprised and shocked many experienced foreign policy commentators, observers of the war on drugs have known for decades that 'fixing the intelligence and the facts on the policy' has been both the very basis and the central flaw of the War on Drugs.

In the lead up to the June 2005 meeting of the Programme Coordinating Board of UNAIDS, over 130 diverse individuals and organisations in the United States of America wrote (see Additional file 2) [8] on May 10, 2005 to Ambassador Randall Tobias, Coordinator of United States Government Activities To Combat HIV/AIDS Globally, 'to express our concern about recent reports that US officials have questioned the efficacy of needle exchange programs and sought to block support for needle exchange in United Nations resolutions and policy documents'. Emphasizing the importance of HIV infection among and from injecting drug users in the United States of America and globally, they noted that 'no fewer than seven federally-funded reviews and reports conducted by public health officials, researchers and US government agencies have concluded that syringe exchange programs are effective, safe and cost effective'. Recent public support for the science of needle syringe programmes was cited including endorsements from the Director of the National Institutes of Health, the Director of the National Institute on Drug Abuse and a recent World Health Organization (WHO) report which stated that the available data 'present a compelling case that needle and syringe programs substantially and cost effectively reduce the spread of HIV among injection drug users and do so without evidence of exacerbating injecting drug use at either the individual or societal level.'

In response, 35 individuals from United States' War on Drugs organizations wrote to Ambassador Randall Tobias on May 25, 2005 (see Additional file 2) [9]. This group lists only six people with medical or other degrees. These 35 individuals claimed to be a 'diverse group of citizens and organizations' who were 'better informed on prevention, intervention and treatment of addiction than any other source'. They urged Ambassador Tobias to 'continue to promote and defend the United States' position against the disease-promoting practices of needle and syringe giveaways'. Although making the remarkable claim that ' [needle syringe] programs are ineffective or, at best, weakly effective at deterring the spread of HIV', no evidence was offered to support this or any of the other propositions offered to Ambassador Tobias.

Fixing 'the intelligence and facts on the policy' has trapped the United States of America into a military quagmire in Iraq and contributed to looming economic problems resulting from the twin current account and Federal budget deficits. Fixing 'the intelligence and facts on the policy' for illicit drugs, ensured tragic health, social and economic consequences for the United States of America [10]. Extending this approach to HIV has magnified these tragic costs. But time is running out: exporting to other countries a failed and futile policy on the twin epidemics of HIV and illicit drugs will soon be a thing of the past. More and more, countries want to fix their drugs and HIV policy on intelligence and facts rather than the other way round.

## Supplementary Material

Additional File 2 *Ambassador Randall Tobias-Zero Tolerance file.doc *A response from 35 individuals and organisations urging Ambassador Tobias to continue to promote and defend the United States' position in a document expressing UNAIDS HIV prevention policy. Click here for file

Additional File 1 *Ambassador Randall Tobias HR.doc *Letter to Ambassador Randall Tobias expressing concern about recent reports that US officials have questioned the efficacy of needle exchange programs and sought to block support for needle exchange in a document expressing UNAIDS HIV prevention policy. Click here for file
