# Supplementary material for: The US war on harm reduction: fixing policy on intelligence and facts
Source: Harm Reduct J. 2005 Sep 15;2:14. doi: 10.1186/1477-7517-2-14 (PMC1262743; doi:10.1186/1477-7517-2-14)
Supplement: Additional File 2 — Ambassador Randall Tobias-Zero Tolerance file.doc A response from 35 individuals and organisations urging Ambassador Tobias to continue to promote and defend the United States' position in a document expressing UNAIDS HIV prevention policy. [file 1477-7517-2-14-S2.doc]

# May 25, 2005

Ambassador Randall Tobias

Coordinator of United States Government Activities

To Combat HIV/AIDS Globally

United States Department of State

2201 C Street, NW

Washington, DC 20522

Via Facsimile

Dear Ambassador Tobias:

The individuals and organizations listed below applaud you and thank you for your commitment to sound public health policy. We urge you to stand firm in your work with the Joint United Nations Programme on HIV/AIDS (UNAIDS) and to continue to promote and defend the United States' position against the disease-promoting practices of needle and syringe giveaways.

We are a diverse group of citizens and organizations that is better informed on prevention, intervention and treatment of addiction than any other source. Our knowledge extends to HIV, AIDS, the hepatitis spectrum diseases, and the role drug addiction plays in their transmission. Among us are educators, advocates, practitioners, physicians, research scientists and policy makers. And as varied as our experiences and opinions, we all agree that it is not good policy for the United States to promote or enable the disease of drug addiction as a strategy to control the spread of HIV or any other infectious process.

The connection between intravenous substance use and HIV transmission is not controversial. What is controversial, for good reason, is the provision of needles to enable intravenous drug use under the guise of HIV prevention. The supposed logic behind needle giveaway programs is that the use of sterile needles helps curb the spread of HIV and other diseases. However, such programs are ineffective or, at best, weakly effective at deterring the spread of HIV. They are even less helpful in deterring the spread of Hepatitis C. Needle giveaway programs wrongly assume that infection occurs in the drug-using population only from contaminated needle sharing. In reality, much of the spread of HIV and other disease among intravenous drug users is the result of high-risk sexual behaviors that are not under inhibitory control when a drug user is under the influence of psychoactive substances.

Moreover, needle giveaway programs are harmful because they normalize, if not encourage, intravenous drug use among non-drug-using, non-dependent drug-using, and drug-dependent citizens alike.

The best way to keep non-drug-using citizens off of drugs is to foster a non-drug using societal norm. But needle giveaways destroy drug prevention efforts by sending a message to children and the community at large that intravenous drug use is a manageable behavior. They foster an incorrect notion that drug addiction is not a disease with deadly consequences.

Needle giveaway programs also harm non-dependent drug users. With pleasurable drug-using experiences and few, if any, consequences, the internal incentives for the non-dependent drug user to stop using are few. External influences are imperative to preventing the non-dependent user from progressing to abuse or dependence. Needle giveaways undermine the non-using norm and reduce external deterrents to drug use by perpetuating the notion that drug use can be controlled.

Taking it one step further, needle giveaways provide the actual tools for drug use. Medical research recognizes that drug-dependent individuals have lost voluntary control over their drug abuse. Whether they want to stop using makes no difference; stopping outright is necessary to treat the disease and ensure the patient's survival.

Needle giveaway programs do not support the cessation of drug use; to the contrary, they enable and perpetuate drug use among the most vulnerable patients – those whose free will has been robbed by deadly drugs of addiction.

The most basic rationale underpinning the United States' policy against needle giveaway programs is this: It is inhumane and counterproductive to deal with two potentially fatal diseases -- intravenous drug use and HIV -- by condoning one in an ineffective effort to prevent the other. A far better strategy is to work vigorously and without ambiguity to deter drug use under all circumstances and to prevent the spread of HIV by promoting full recovery for those suffering from abuse and dependence.

On a final note, Ambassador Tobias, we know firsthand what formidable foes drug legalizers can be. If ever we can be of support or assistance to you -- by providing research findings, expert testimony, or a mere reminder of our gratitude for your commitment to sound public health policy -- please call on us.

Sincerely,

Andrea G. Barthwell, M.D., F.A.S.A.M.

Founder and CEO

EMGlobal LLC

Global Public Health Policy

1010 Lake Street

Suite 422

Oak Park, IL 60301

Betty S. Sembler

Founder and Chair

Save Our Society From Drugs

2600 9th Street North

Suite 200-A

St. Petersburg, FL 33704

Robert B. Charles

Former Assistant Secretary for

International Narcotics and Law

Enforcement Affairs

United States Department of State

The Charles Group

18630 Reliant Dr.

Gaithersburg, MD 20879

Judy Kreamer

President

Educating Voices, Inc.

P.O. Box 6084

Naperville, IL 60567

Calvina L. Fay

Executive Director

Drug Free America Foundation, Inc.

2600 9th Street North

Suite 200-B

St. Petersburg, FL 33704

Robert L. DuPont, M.D.

First Director

National Institute on Drug Abuse

President

Institute for Behavior & Health, Inc.

6191 Executive Boulevard

Rockville, MD 20852

David G. Evans, Esq.

Executive Director

Drug-free Schools Coalition

203 Main St., PMB 327

Flemington, NJ 08822

Eric Voth, M.D., F.A.C.P.

Chair

Institute on Global Drug Policy

901 Garfield

Topeka, KS 66606

David A. Gross, M.D.

Chair

International Scientific and Medical

Forum on Drug Abuse

4800 Linton Blvd.

Bldg. D, #503

Delray Beach, FL 33445

DeForest Rathbone

Chairman

National Institute of Citizen Anti-drug

Policy (NICAP)

Public Drug Policy Advocates

1044 Springvale Rd.

Great Falls, VA 22066

Susie Dugan

Executive Director

PRIDE-Omaha, Inc.

3534 South 108 Street

Omaha, NE 68144

John F. Gilligan, Ph.D.

President Emeritus

Fayette Companies

600 Fayette St.

Peoria, IL 61654

Peter B. Bensinger

President & CEO

Bensinger, DuPont & Associates

20 N. Wacker Drive, Ste. 920

Chicago, IL 60606

Jeanette McDougal, MM, CCDP

Director

National Alliance for Health and Safety

P.O. Box 54893

Jacksonville, FL 32245

Michael C. Barnes, Esq.

Managing Attorney

DCBA Law, PLLC

International Law and Public Policy

P.O. Box 2943

Leesburg, VA 20177

John Pastuovic

Founder and President

John Pastuovic Communications, Inc.

Public Interest Communications

117 Oneida Street

Elmhurst, IL 60126

Anne Meyer

Secretary

Educating Voices, Inc.

P.O. Box 6084

Naperville, IL 60567

Jessica Talbert

Policy Analyst

Just Good Policy

2000 South Eads St.

Arlington, VA 22202

Terrence P. Farley

First Assistant Prosecutor, Director

Ocean County Strike Force

119 Hooper Ave.

Toms River, NJ 08753

Joyce D. Nalepka

Drug-Free Kids: America's Challenge

Nat'l Network of Parents/Grandparents

6213 Executive Boulevard

Rockville, MD 20852

Robert M. Stutman

Former DEA Special Agent in Charge

Member of the Board

Educating Voices, Inc.

P.O. Box 6084

Naperville, IL 60567

Roger Morgan

Founding Chairman

Coronado SAFE Foundation

5771 Sweetwater Rd.

Bonita, CA 91902

Carla Lowe

Co-founder

Californians For Drug-Free Schools

4241 Rio Monte Court

Carmichael, CA 95608

Debbie Lindner, President

New Jersey Federation for Drug Free

Communities

Statewide Non-Profit Prevention

Organization

P.O. Box 702

Livingston, NJ 07039

Geraldine Silverman, Chairman

Millburn Municipal Alliance for Drug

Awareness

Community-Based Prevention

Organization

23 Audubon Court

Short Hills, NJ 07078

William R. Caltrider, Jr.

President

Center for Alcohol and Drug Research

and Education

6200 N. Charles Street

Baltimore, MD 21212-1112

Malcolm "Cap" Beyer

Chairman

Student Drug-Testing Coalition

92 Lighthouse Drive

Jupiter, FL 33469

Elizabeth Edwards

Community Advocate

for Drug-Free Schools, Workplaces and

Communities

5442 E. 6th St.

Tucson, AZ 85711

Theresa Costello

Port Richmond Community Group

c/o 3117 Tulip St.

Philadelphia, PA 19134

Audrey Bumanis

Research Associate

Institute for Behavior & Health, Inc.

6191 Executive Boulevard

Rockville, MD 20852

Judy Dinerstein

Board Member

Educating Voices, Inc.

P.O. Box 6084

Naperville, IL 60567

Sandra Bennett

Director

Northwest Center for Health and Safety

419 E. Cedar Street, Ste. A 209

LaCenter, WA 98629

Linda Ledger, Vice-President

New Jersey Federation for Drug Free

Communities

Statewide Non-Profit Prevention

Organization

P.O. Box 702

Livingston, NJ 07039

Herschel Mills Baker

President

Australian Parents for Drug Free Youth

P.O. Box 73

Maryborough Queensland

Australia 4650
