# Supplementary material for: The US war on harm reduction: fixing policy on intelligence and facts
Source: Harm Reduct J. 2005 Sep 15;2:14. doi: 10.1186/1477-7517-2-14 (PMC1262743; doi:10.1186/1477-7517-2-14)
Supplement: Additional File 1 — Ambassador Randall Tobias HR.doc Letter to Ambassador Randall Tobias expressing concern about recent reports that US officials have questioned the efficacy of needle exchange programs and sought to block support for needle exchange in a document expressing UNAIDS HIV prevention policy. [file 1477-7517-2-14-S1.doc]

Ambassador Randall Tobias

Office of the United States Global AIDS Coordinator

US State Department

# 2201 C Street NW

Washington DC 20522

Dear Ambassador Tobias:

As the US increases its commitment to global HIV prevention efforts, we write to express our concern about recent reports that US officials have questioned the efficacy of needle exchange programs and sought to block support for needle exchange in United Nations resolutions and policy documents. As you know, the sharing of syringes by injection drug users is a major driver of the AIDS epidemic both in the United States and internationally. As researchers, practitioners and representatives of affected populations, we affirm the important role that needle exchange and other sterile syringe programs play in helping to avert the spread of HIV/AIDS.

Outside of Africa, an estimated one-third of new HIV infections are attributed to injection drug use. In some countries, the proportion is even higher. In Russia, as many as 1.2 million people are living with HIV, with 75 percent of new infections stemming from injection drug use. Injection drug use accounts for the majority of HIV/AIDS cases in Ukraine, all of the Baltic States, the Central Asian Republics, Indonesia, Nepal, Iran, and Pakistan. In China, Vietnam and Malaysia, the majority of those living with HIV are injection drug users. Often, the vast majority of those infected are under thirty. Injection driven HIV epidemics consistently spread faster than sexually transmitted epidemics such as those found in sub-Saharan Africa.

In the United States, according to the Centres for Disease Control and Prevention, 23 percent of new AIDS cases reported in 2003 were attributed to injection drug use and/or sex with an injection drug user. This percentage is higher for women and African American men. Of all cases diagnosed through 2003, 53 percent of cases among women and 43 percent among African American men were attributed to injection drug use or sex with an injection drug user.

As the US Public Health Service has noted, for injection drug users who cannot or will not stop using drugs, using sterile syringes remains the safest and most effective method of HIV prevention. Medically appropriate treatment remains out of reach to the vast majority of drug users who need it: in the United States, one of the world's richest countries, over 80 percent of those needing treatment for drug abuse as of 2000, did not have access to it. At the same time, extensive evaluation and research has shown that sterile syringe programs can dramatically decrease the spread of HIV without increasing drug use. These programs can additionally provide a bridge to drug treatment services by offering clients with information and referrals to treatment providers. No fewer than seven federally-funded reviews and reports conducted by public health officials, researchers and US government agencies have concluded that syringe exchange programs are effective, safe and cost effective.

The efficacy of syringe exchange has recently been affirmed by the US government's top scientists. In an October 7, 2004 letter to Congress, Director of the National Institutes of Health Dr. Elias Zerhouni stated, "Research shows that SEPs [syringe exchange programs], when implemented as part of a comprehensive HIV/AIDS prevention strategy, can be an effective public health approach to reduce the spread of HIV and other blood borne pathogens in the community." In an August 4, 2004 letter, Director of the National Institute on Drug Abuse Dr. Nora Volkow stated, "The majority of studies have shown that NEPs/SEPs [needle exchange programs/syringe exchange programs] are strongly associated with reductions in the spread of HIV when used as a component of comprehensive approach to HIV prevention. In addition to decreasing HIV infected needles in circulation through the physical exchange of syringes, most NEPs/SEPs are part of a comprehensive HIVprevention effort that may include education on risk reduction, and referral to drug addiction treatment, job or other social services, and these interventions may be responsible for a significant part of the overall effectiveness of NEPs/SEPs. NEPs/SEPs also provide an opportunity to reach out to populations that are often difficult to engage in treatment."

In 2004, this consensus was reinforced by the World Health Organization (WHO), which stated that the available data "present a compelling case that needle and syringe programs substantially and cost effectively reduce the spread of HIV among injection drug users and do so without evidence of exacerbating injecting drug use at either the individual or societal level." The WHO accordingly recommended that "authorities responsible for areas threatened by or experiencing an epidemic of HIV infection among IDUs (injection drug users) should adopt measures urgently to increase the availability and utilization of sterile injecting equipment and expand implementation to scale as soon as possible."

As the single largest funder of global HIV/AIDS programs in the world, the US carries enormous influence in shaping global approaches to the epidemic. Even beyond its role as a donor, many look to the United States for guidance in the design and implementation of their programs. The fact that the United States remains the only country in the world to impose an explicit ban on federal funding of needle exchange already sets a poor example

to other nations battling severe injection-driven HIV/AIDS epidemics. It is more critical than ever that US agencies communicate accurate information about needle exchange, faithfully represent the public health consensus supporting these programs, and refrain from standing in the way of countries, and organizations within its own borders, that wish to pursue this proven, effective method of prevention. With so many at risk for

HIV, we need all the tools we have.

Sincerely,

Access Works, Minneapolis, MN

Advocates for Recovery through Medicine, MI

African Action, Washington, DC

African Services Committee, New York, NY

After Hours Project, Brooklyn, NY

AIDS Action Baltimore, Baltimore, MD

AIDS Alliance for Children, Youth & Families, Washington, DC

AIDS Center of Queens County, Queens, NY

AIDS Foundation of Chicago, Chicago, IL

AIDS Institute, Washington, DC

AIDS Mastery Fund, New York, NY

AIDS Project Los Angeles, Los Angeles, CA

AIDS Services of Austin, Austin, TX

AIDS Survival Project, Atlanta, GA

AIDS Treatment Activists Coalition, New York, NY

AIDS Vaccine Advocacy Coalition, New York, NY

Alcoholism and Addiction Outpatient Program,

Saint Vincent Catholic Medical Center - New York, NY

Alliance for Inmates with AIDS, New York, NY

American Academy of HIV Medicine, Washington, DC

American Jewish World Service, New York, NY

Americans for Safe Access, Oakland, CA

amfAR, The Foundation for AIDS Research, New York, NY

Bailey House, New York, NY

Beth Israel Medical Center, New York, NY

Bexar Area Harm Reduction Coalition, San Antonio, TX

Center for Health and Gender Equity, Takoma Park, MD

CHAMP, New York, NY

Chicago Recovery Alliance, Chicago, IL

CitiWide Harm Reduction, Bronx, NY

Crane Center, Santa Clara Public Health Dept, Santa Clara, CA

DanceSafe NYC, New York, NY

Doctors for Global Health, Decatur, GA

Dora Weiner Foundation, Staten Island, NY

Douglas County HCV Task Force, Roseburg, OR

Drug Overdose Prevention and Education Project, San Francisco, CA

Drug Policy Alliance, New York, NY

Elton John AIDS Foundation, New York, NY

Empire State Coalition of Youth and Family Services, New York, NY

Foundation for Integrative AIDS Research, Brooklyn, NY

Foundation for Research of Sexually Transmitted Diseases, New York, NY

Global AIDS Alliance, Washington DC

Gay Men's Health Crisis, New York, NY

Harm Reduction Coalition, New York, NY

Harm Reduction Project, Salt Lake City, UT

Harm Reduction Psychotherapy and Training Associates, New York, NY

Harm Reduction Therapy Center, San Francisco, CA

Health Alliance International, Seattle, WA

Health Equity Project, New York, NY

Health Gap (Global Access Project), Washington, DC

Health Initiatives for Youth, San Francisco, CA

Health Science Department of City College of San Francisco, San

Francisco,CA

HIV Advocacy Council of Oregon & SW Washington, Portland, OR

HIV Center for Comprehensive Care, St. Luke's-Roosevelt Hospital, New

York, NY

HIV Education and Prevention Project of Alameda County/Casa Segura

Oakland, Ca

HIV Law Project, New York, NY

HIV Medicine Association, Alexandria, VA

HIV Resource Center, Roseburg, OR

HIV/HCV Committee of California Prison Focus, San Francisco, CA

Housing Works, New York, NY

Human Rights Campaign, Washington, DC

Human Rights Watch, New York, NY

Institute for Behavior and Health, Rockville, MD

Interfaith Drug Policy Initiative, Washington, DC

International Community of Women Living with HIV/AIDS, Washington, DC

International Women's Health Coalition, New York, NY

Intersect-Worldwide, New York, NY

Jovenes 24 Hrs A.D., New York, NY

JRI Health, Boston, MA

League of Pissed Off Voters, New York, NY

Lifelong AIDS Alliance, Seattle, WA

Lower East Side Harm Reduction Center, New York, NY

Maine HIV Advisory Committee, Portland, ME

Mano a Mano, New York, NY

Marin Services for Women, Greenbrae, CA

Michigan Positive Action Coalition (MI-POZ), Detroit, MI

Midwest Harm Reduction Institute, Chicago, IL

National Advocates for Pregnant Women, New York, NY

National Alliance of State & Territorial AIDS Directors, Washington, DC

National Association of People with AIDS (NAPWA-US), Washington, DC

National Association of Social Workers, Washington, DC

National Development and Research Institutes, New York, NY

National Lawyers Guild-Loyola New Orleans School of Law Chapter, New

Orleans, LA

National Minority AIDS Council, Washington, DC

NEED, Berkeley, CA

Network of Sex Work Projects, New York, NY

New Mexico AIDS Service, Albuquerque, NM

New York Academy of Medicine, New York, NY

The New York City Association of Homeless and Street-Involved Youth

Organizations, New York, NY

NM POZ Coalition/Planetpoz, Santa Fe, NM

North American Syringe Exchange Network, Tacoma, WA

Open Society Institute, New York, NY

Pangaea Global AIDS Foundation, San Francisco, CA

Partnership Project, Portland, OR

PartySmart, Northern NM Chapter of Dancesafe, Los Alamos, NM

Physicians for Human Rights, Cambridge, MA

Physicians for Human Rights, Washington, DC

Points of Distribution, Oakland, CA

Point Defiance AIDS Project, Tacoma, WA

Positive Health Project, New York, NY

POZ Magazine, New York, NY

Presbyterian Church (USA) Washington Office, Washington, DC

Prevention Point Pittsburgh, Pittsburgh, PA

Prevention Works, Washington, DC

Priority Africa Network, Berkeley, CA

Project BUILD of Richmond County, Staten Island, NY

Project Inform, San Francisco, CA

Public Citizen's Health Research Group, Washington, DC

Redwood AIDS Information Network and Services, Redway, CA

Responsible Recovery, Emeryville, CA

Roseburg Risk Reduction, Roseburg, OR

Roseburg VA Medical Center, Roseburg, OR

San Antonio AIDS Foundation, San Antonio, TX

San Francisco AIDS Foundation, San Francisco, CA

San Mateo AIDS Program, San Mateo, CA

SENSEI Health, New York, NY

Southern AIDS Coalition, Birmingham, AL

Southern Tier AIDS Program, Johnson City, NY

Street Health Works, San Francisco, CA

Street Works, Nashville, TN

Student for Sensible Drug Policy, Washington, DC

Student Social Workers Alliance for a Progressive

Society, New York, NY

Tassin's Healthy Alternative's Group, Chicago, Il

Test Positive Aware Network, Chicago, IL

Title II Community AIDS National Network, New York, NY

Treatment Action Group, New York, NY

Unitarian Universalist Association of Congregations, Boston, MA

United Church of Christ, Cleveland, OH

Utah AIDS Foundation, Salt Lake City, UT

Vermont Harm Reduction Coalition, VT

The Well Project, Charlottesville, VA

Women's Equity in Access to Care and Treatment for HIV/AIDS Initiative

(WE-ACTx), San Francisco, California
